# 

**Published:** 1911-03-15

**Authors:** 


### ANNOUNCEMENTS.

### ILLINOIS STATE DENTAL SOCIETY.

The forty-seventh annual meeting of the Illinois State Dental Society will be held at Peoria, May 9, 10, 11 and 12, 1911. J. P. Luthringer, chairman local arrangements committee, Peoria; J. F. F. Waltz, secretary, Decatur.

### ALUMNI ASSOCIATION OF THE ST. LOUIS DENTAL COLLEGE.

The Annual Clinic of the Alumni Association of the St. Louis Dental College will be held in the college building on Saturday, April 22, 1911. All ethical practitioners are cordially invited to attend. Fancis P. Mahon, secretary.

### INDIANA STATE DENTAL ASSOCIATION.

The fifty-third annual meeting of the Indiana State Dental Association will be held at Indianapolis, May 16, 17 and 18, 1911, at the Claypool Hotel. Plans are being perfected for a great meeting. C. D. Lucas, president, Indianapolis, Ind., Otto U. King, secretary, Huntington, Ind.

### SOUTHERN BRANCH NATIONAL DENTAL ASSOCIATION

A complimentary dinner will be given by the members of the Southern Branch of the National Dental Association during its meeting in Atlanta, Ga., April 4, 5 and 6, to Dr. Frank Holland, of Atlanta, Ga.

Thos. P. Hinman, Chairman.

### THE KENTUCKY STATE DENTAL ASSOCIATION.

The forty-second annual meeting of the Kentucky State Dental Association will be held at Owensboro, May 25, 26 and 27, 1911. This meeting is to be the last state dental society to hold its annual meeting, according to the circuit between Iowa, Illinois, Indiana and Kentucky; hence we expect to have an unusually large exhibit, as well as a good program. All members of the profession are cordially invited. W. M. Randall, Secretary.

### ALUMNI ASSOCIATION, WASHINGTON UNIVERSITY DENTAL SCHOOL.

A reunion and clinic of all graduates will be held under the auspices of the Alumni Association of Washington University Dental School, April 10 and 11, at the college building, Twenty-ninth and Locust streets. An unusually interesting program is in preparation and the meeting promises to be the best in the history of the school. All graduates should mark the dates in their appointment books and make special effort to be present. They will be repaid for the time spent. All ethical dentists are invited to attend.

R. A. Harris, E. A. Woelk and W. H. Scott, publicity committee.

### INSTITUTE OF DENTAL PEDAGOGICS.

At the annual meeting of the Institute of Dental Pedagogics, held in Washington, D. C., December 27, 28, and 29, 1910, the following officers were elected for the ensuing year: President, Dr. Donald M. Gallie; vice-president, Dr. H. E. Friesell; secretary-treasurer, Dr. Fred W. Gethro, 917 Marshall Field Building, Chicago. The next meeting will be held in Chicago and the time has been changed to one month later, the dates being January 24, 25 and 26, 1912.

Fred W. Gethro. Secretary-Treasurer.

---

NATIONAL DENTAL ASSOCIATION—SOUTHERN BRANCH.

The Southern Branch of the National Dental Association will meet in Atlanta, Ga., April 4, 5 and 6 at 10:00 a. m. Headquarters will be at the Piedmont Hotel. All business sessions will be held there, and the manufacturer's exhibit also. The railroads have given the following rates: A fare and one-half plus fifty cents, in all territory south of the Mason and Dixon line, and east of the Mississippi, provided there are two hundred attendants who travel by rail. Those living outside of this direct will please communicate with the corresponding secretary, Dr. W. G. Mason, Tampa, Fla., as to rates attainable. The various committees are hard at work, and the meeting promises to be an exceptionally good one. The time selected for the meeting is ideal in this section, and as it is well in advance of the commencement exercises, it is expected that we will have an unusually large attendance. A more complete announcement will be made in next month's journals, but it is well to make your other arrangements fit in with these dates.

C. M. Barnwell, Jr.,
Chairman of Committee on Arrangements.

---

THE HYGIENE OF THE TEETH.

(By Dr. Schaeffer-Stuckert, Frankfort-on-Main.)

The International Hygiene Exhibition, Dresden, 1911, has assigned a special department, with practical and scientific exhibits, to "Diseases of the Teeth." Thus due and gratifying recognition is paid to the hygiene of the teeth, that young branch of dental pathology and practice which is making vigorous growth. Although the hygienic efforts of dental surgeons and their active care for the general welfare of the people are of comparatively recent date, the representatives of dental hygiene have achieved success that deserves to be demonstrated in an international exhibition for hygiene. Thr chairmanship of the department "The Diseases of the Teeth," has been accepted by Professor Dr. Walkhoff; the vice chairman will be: Professor Dr. Dieck, Berlin; Professor Dr. Jessen, Strasburg, I. E.; and Dental Surgeon Dr. Schaeffer-Stuckert, Frankfort-on-Main. The program of the department will embrace everthing that comes under the head of, or has reference to, the hygiene of the teeth; the object being to give a scientific and complete survey of the importance of the surgeon-dentist's sphere of work. This program will be conveniently arranged in two principal parts. In the first the visitor will find ocular demonstration of the causes and consequence of bad teeth; in the second he will be shown to what extent and in what way decay of the teeth which is a widespread folk-disease, can be combated, and how far hygienic science, as applied to the teeth, has hitherto worked advantageously.

The causes of bad teeth are manifold, and closely connected with questions of general hygiene, of the care of the body of food hygiene, and cognate matters. Here, therefore, side by side with the ocular demonstration of the development of the jaws and teeth and of their finer structure, will be seen the pathological appearances that are attributal to faulty development and illness or weakness in general. Here also opportunity will be afforded of showing the value of "orthodonty" (the regulation of teeth and jaws) which has become so important in late years.

Reciprocally, however, the diseases of the teeth react injuriously on the human organs which hygiene sets itself to keep in a sound condition. Therefore the combating of decay in the teeth—or, as dentists call it, the keeping the mouth healthy—must be described as one of the measures, or means to an end, of hygiene. Stomach and bowel diseases, tuberculosis, and infection in general, are among the after-consequences of carries.

The special province of the hygiene of the teeth is to combat the wide-spread and erroneous idea that the health or ill-health of the mouth is of secondary interest, and to prove that the condition of each individual mouth is not a matter of indifference to hygiene in general. Dentists have naturally recognized first their social mission, and do not consider that combating decay of the teeth and its consequences, still less that scientific inquiry into its causes and into the extent of the evil, are their tasks. It is here that the names Rose, Jessen, Michel, Lenhardtsen, Godon, Roy, Cunningham, and others call for particular mention. It is in Germany in particular that the hygiene of the teeth has actually found a footing. The statistics of the extent to which caries prevails will be made clear to the vision of beholders.

Those statistics it was that ultimately induced dental surgeons to place the combating of caries on a broad basis. The arrangement of school dental hospitals, the introduction of dental surgery into the army and navy, the winning over of financial authorities to the cause of dental treatment, the provision of expert dental practitioners for hospitals, asylums, prisons, holiday colonies, etc.,—that is the special province of the hygiene of the teeth. By means of plans, drawings, reports, and illustrated lectures, visitors to the exhibition will obtain a comprehensive view of what is already being done in this direction in civilized states. Germany has already accomplished fruitful results through the committee for the care of the teeth in schools, under the chairmanship of his Excellency von Moller, retired minister of state, Dental-surgical aid for the children attending the elementary public schools has been secured in many towns. School dental hospitals have been provided in Strasburg, Darmstadt, Koln, Berlin, Frankfort-on-Main, Ulm, Mulhausen, and many other places.

The "Diseases of the Teeth" department in the Dresden International Exhibition should, in addition to supplying a great number of examples, promote the universal appreciation of dental hygienic aims, and stimulate many to imitate and emulate those examples. Not only will this young plant be shown to visitors to the exhibition, and to the leaders and their followers in other departments, the recognized exponents of general hygiene, but the will and purpose will be expressed to co-operate in the great work, hygiene, the maintenance of the health of the people.

This is also to say, that the hygiene of the teeth will be further demonstrated in other departments: whether in separate pavillions, or in school hygiene, military sanitation or the care of the sick in towns.

N. B.—Application for space in the department "Hygiene of the Teeth" should be made as follows: From Germany, to Dr. Dieck, Berlin W. Lutzow Strasse; from abroad, to Direktor Dr. Schaeffer-Stuckert, Frankfort-on-Main, Kettenhofweg, 29.

---

The New Jersey State Board of Registration and Examination in Dentistry will not hold their semi-annual examination in the Assembly Chamber of the State House Trenton, N. J., June 26th, 27th, 28th, 1911.

All applications must be in the hands of the secretary ten days prior to the examination.

For information address; Charles A. Meeker, D.D.S.
29 Fulton St., Newark, N.Y.

---

NEW JERSEY STATE DENTAL SOCIETY.

The forty-first annual meeting of the New Jersey State Dental Society will convene at Asbury Park, N. J. in the

Casino on the 19th., 20th., and 21st. of July 1911, beginning Wednesday at 10 A. M.

Extensive alterations will be made on the main floor and the largest exhibit ever given by any State Society will be inaugurated showing modern dental furniture and up to date instruments.

Application at an early date to the chairman of the exhibit committee, Dr. W. W. Hawke of Flemington, N. J. is necessary to secure space.

Papers and Clinics by the most eminent men of the profession.

Watch the Dental Journals for further particulars.

Charles A. Meeker, D.D.S., Sec'y.

---

MEETING OF THE TEXAS STATE DENTAL ASSOCIATION.

The thirty-first annual meeting of the Texas State Dental Association will be held in San Antonio May 11, 12, and 13.

A cordial invitation is extended all ethical members of the profession.

J. G Fife, Sec'y.
Dallas Texas.

